# How can attending physicians be more attentive? On being attentive versus producing attentiveness

**DOI:** 10.1007/s11019-015-9669-y

**Published:** 2016-03-07

**Authors:** Klaartje Klaver, Andries Baart

**Affiliations:** 1 Department of Culture Studies, Tilburg University, Tilburg, The Netherlands; 2 Optentia Research Focus Area, North-West University, Vanderbijlpark, South Africa

**Keywords:** Attentiveness, Emergence, Indefiniteness, Ethics of care

## Abstract

This article is about caregivers being attentive to patients in healthcare. From earlier work on the understanding of the other, we know that it is impossible to completely understand the experiences of others. By the sharing of subjectivity—intersubjectivity—we may try to ‘grasp’ the other’s point of view. However, we can never assume that the *same* experience produces the same *experience*. Now, if it is principally impossible to understand the experience of one another, and if paying attention always implies an understanding of what to pay attention to, then how is it possible to be attentive to the experiences of those who are entirely at the mercy of our care? How can caregivers perceive the impossibility of understanding the experiences of patients as an appeal to be attentive to their experiences? This is discussed in this article. It departs from the authors being confronted with inexplicabilities in the empirical study of attentiveness in healthcare. It presents two examples and discusses the meaning of these emergent properties. This leads to a discussion of the existent literature on the indefiniteness and openness of attentiveness. It becomes clear why, although we can understand and predict much of it, attentiveness will always be characterized by a certain uncontrollability as well.

## Introduction

Paying attention is a process of directed observation of the environment. It has different characteristics, but it always involves observation, or perception, and interpretation (Arvidson 2006). If you are paying attention to something, you take something *as something*. For example, you see something circular as a ball. Or as a balloon. Or as the belly of your pregnant friend. What you perceive is not fixed, but it is meaningful and thus an interpretation of what occurs. Your attention to the balloon is associated with understanding that what you see is a balloon. What you feel does also play a role in attentiveness. Your attention to your pregnant friend’s belly, for instance, is associated with your joy about the fact that she is expecting a child.

This also applies to care. For example, your attention may be drawn to a patient who is crying. However, you do not know what that crying means. Maybe you can find it out by asking, but that is not always possible, due to the circumstances or because the patient does not tell you what the matter is. Then what? In practice, this often means that the caregiver’s attentiveness moves away and focuses on something he can do something about or something he can understand instead (Klaver and Baart 2016).

We, caregivers, have to understand patients. Interest in the patient’s experience is growing in all facets of health care, which is shown by the increasing number of health care institutions making ‘patient experience’ a strategic goal, insurance companies that want to gain insight in the quality of the patient experience to rely their policy on, the increasing amount of ‘lifeworld studies’, and so on. In the healthcare sector, mainly dominated by the medical profession, it becomes more and more clear that we not only need to understand about diseases, but also about the people who suffer them. The notion of ‘patient-centeredness’ has increasingly influenced healthcare in the Netherlands and elsewhere. Patient experience has been given high priority. In the midst of consumer driven concerns, the aim has been to give patients more ‘voice and choice’ in their own health care (Berwick 2009).

Patient experiences have not always been a central value in the health care practice, since caregivers must pursue other values, such as strictly working according to protocols, finishing tasks in time, meeting production standards, or showing in a good light amongst colleagues. The experiences of patients are often moved to the background. Therefore, every patient’s story, every study or other attempt to come closer to the experience of a sick person, is a major victory. However, since there are as many stories as there are people, the experience of one patient never speaks for another one. Patient experiences have to do something impossible, but something that is impossible to ignore as well: they must speak on behalf of others whose experience we do not know.

In fact, generally speaking, it is impossible to understand the experience of a patient. When trying to ‘grasp’ the patient’s point of view, sharing experiences with them seems the most appropriate approach. The sharing of subjectivity—intersubjectivity—creates moments of recognition and the intuition that we have ‘grasped’ the other’s point of view. At the same time, however, we can never assume that the *same* experience produces the same *experience* (Van der Geest 2007: 9; our italics). This refers to the problem of identification (Gadamer in Fay 1996: 8–50). Gadamer would say it is essential for the understanding of an experience that the observer must be able to identify with the experience. He needs to understand it as a particular experience and assess it like that. The observer should be able to identify the experience; this identification makes it an experience. Another essential aspect of understanding an experience is that the identity of that experience can be understood only if the observer ‘plays along’. According to Gadamer, the real understanding of an experience can only be achieved if there is an active attitude that establishes a meaning. This makes the observer a fellow actor in the experience. An experience supposedly consists of two aspects: the experience itself and the observer who plays along in the game of the experience. This makes it intrinsically impossible to understand the experience of the other in the same way as the other does.

Now, if it is principally impossible to understand the experience of one another, and if paying attention always implies understanding, then how is it possible to be attentive to the experiences of those who are dependent on our care? How can caregivers perceive the impossibility of understanding the experiences of patients as an appeal to be attentive to their experiences? This is discussed in this article.

## Two propositions

As this paper is about care and attentiveness, it must be clear what our view of care involves. In this view of care, attentiveness is a core issue. Our approach includes two main assumptions. These assumptions are common in care ethics, the theoretical approach in which our thinking is placed. The first premise is that care takes place in relationships. Persons, communities, and organizations are conceptualized as relational and interdependent (Held 2006; Van Heijst 2011).

The second premise is that care is always context-bound and situation-specific (Tronto 1993). One can discern three forms of context: the physical context such as the place where you live, the social context that assumes that everyone is in a relational network, and the historical context that takes into account someone’s biography (Klaver et al. 2014).

## Background of the problem: inexplicabilities in the study of attentiveness in health care

Because attention is an essential element in good care and at the same time lacks a single definition, we conducted a qualitative empirical research. This study yielded a grounded model that describes different types of attentiveness and explains its occurrence (Klaver and Baart 2016). The analysis showed that a descriptive model of attentiveness comprises a coherent set of the clusters perception (A), object finding (B), and space for attentiveness (C). Our data show nine types of attentiveness. We answered the question why a caregiver practices one type of attentiveness in a certain situation, and not another type. First, it appeared to be of crucial importance whether attentiveness is essential for giving care in the opinion of the caregiver. Second, the focus of attention is essential. Care given by doctors and nurses is always ambivalent; on the one hand, it concerns the body, and on the other hand, it involves the person whom that body belongs to (ibid.).

During our empirical research, we have also found that, at the same time, attentiveness always seems to escape the analysis partly. Although we can identify what factors are of influence, there is still something in the emergence and the nimbleness of attentiveness which we cannot grasp. These inexplicabilities coming forward in the analysis, is the reason for this paper.

In the analysis we have described different types of attentiveness and we have seen how these emerged. We have looked at complete cases, i.e. from the emergence of attentiveness to its outcome for the patient. This means that, in the analysis of the empirical data, the effect of the attentiveness is included in the nomination and description of an attention type. We have described the various factors that have affected the outcome. Yet this is not a process of cause and effect which can be applied reversely as well. We found that even if the influencing factors are the same, another type of attentiveness with another result may emerge. Even though we had a good view of the variables and we could quite well understand why a certain kind of attention had occurred, it appeared that the type of attentiveness was not entirely predictable. The explanation of attentiveness was deficient.

For example, the circumstances may be structured in such a way that based on what we have learnt about attentiveness, the attention is expected to be very brief and focused, and nevertheless, the caregiver may suddenly perform attentiveness of an open kind. Apparently, the caregiver did experience the need and space to be attentive in an open way, while this was not the case in similar situations.

From the analysis of our empirical data, it became clear that the occurrence of attentiveness is always associated with something unpredictable and not exhausted by the empirically shown mechanisms. This was the starting point of this paper. In the literature, we found that these unpredictable aspects are described as the result of emergence. Emergent properties (Johnson 2006; Sawyer 2003; Rehder 2003) can be thought of as unexpected, unaccountable, and untraceable behaviours that stem from interaction between the components of a phenomenon and their environment. It seemed that, although attentiveness can be understood to a large extent, there is always a moment that escapes the prediction. Many factors can be explained but at the same time attentiveness will always be characterised by a certain uncontrollable aspect. This paper departs from the finding that attentiveness has to do with a layered causality, and it will show that this implies that a certain irreducibility and unpredictability are to be included in the analysis.

## Emergence on the level of the caregiver

Emergent properties are explained above as unavoidable elements in the complex practices of attentiveness. In this section, we will extend this idea and propose that this inexplicable nature is not only an unavoidable element but also an indispensable ingredient of good attentiveness—and therefore, there should be space for it in healthcare.

As we have shown, when analysing the data, we as researchers knew what the effect of the attentiveness had been, and therefore we gave a certain type of attentiveness a certain name. Thus, the effect of attentiveness is included in the understanding of the type occurred. However, the caregiver does *not* know in advance which type of attentiveness is going to appear.^1^

In the cases of the more ‘open’ types of attentiveness, when the attentiveness is not (yet) or not exclusively focused on one object, the caregiver often makes a guess and they do something which is not directly deducible to a concrete goal, or they refer to something they cannot quite predict or control. This is what we call emergence on the level of the caregiver. We will illustrate this by means of examples. We present two case descriptions from our study, and then explain that emergent properties seem to be at work.

The first example is about a physician-assistant who has a very stressful day. As his colleague is ill, he must visit patients on other wards and also help out in the emergency department. In the afternoon, he does his round on his own ward. A visit to this patient was not planned, but a nurse asks the doctor to. The patient is a man with cancer in an advanced stage who has trouble eating. He is sitting on his bed in T-shirt and underpants. There are flowers on his bedside table and children’s drawings on the wall. The man has a frolic, round face and a big belly. He is worried about not eating well. “I used to be a gourmand, as they call it. But there is little gourmand left”, the patient says. The doctor replies: “Do you mean you are throwing up all the time, or that nothing tastes good to you anymore?” What follows is a discussion about optimizing the situation under all circumstances. It covers the patient’s perception of the situation. The doctor is aware of the medical problems that have to do with eating, but he also has an eye for the wider, existential experience of the patient. By listening to the utterance of the patient, to the words he chooses, and by not only asking for the things relevant to the medical treatment, he leaves room for the perspective of the patient’s experience to open up. Eventually, the case turns out not to be about having problems with eating food, but about being less able to enjoy life.

Another example is about a lady who has recovered from cancer and now visits the oncologist twice a year for a check-up. She is a rather opinionated woman who takes little note of the advice of the doctor. She also laughs at her husband who is trying to influence her health behaviour through the oncologist. What we see is that the woman is playing with the doctor. She lies and cheats, and does not listen to him. In a sense, the patient exerts force on the doctor. However, the doctor continues to receive and see her. He plays along with her and listens to her little lies. Eventually, it all turns out to be about faith and loyalty.

The attentiveness that has occurred in the above cases, is of two different types. In the first case, the attentiveness is relational, which means that there is no preset goal, but what is at stake for the patient emerges in the conversation and the doctor responds accordingly. This is remarkable because the doctor is very busy and actually had other plans. The question he asks is in line with his stressful day: not quite open; however, it works out well. Our data show that in similar cases, there usually occurs at most a very focused, framed attention. In the second case, the attentiveness ‘condones’. The doctor allows the patient to play with him a little. In retrospect, it appears that space has arisen for what is currently the most important for the patient, namely that she does the most necessary in order to stay healthy, and that she visits the oncologist for her semi-annual checkups.

In both cases, a different kind of attentiveness rises than we would expect based on the grounded theory (Klaver and Baart 2016). As described earlier, the emergence of a certain type of attention is more than the sum of its parts. There will always be unpredictable parts, both for the caregiver and the researcher. These emergent properties originate from the interaction of the caregiver and the patient. In the relationship between them, things can come into existence that cannot be reduced to just either of them (Klaver and Baart 2011). Secondly, the environment affects what may emerge as well (ibid.). In this study, the field (locus) of the emergence is the caregiver. Properties that could not be predicted may arise from their interaction with the patient and the contextual factors.

Based on the data, we can distinguish between emergence on the level of perception and emergence on the level of social interactions.^2^ The first stems from the operation of consciousness, perceptions, intentionality, reviews, moral sensitivity, etc. while the second is associated with work culture, the functioning of the team, the patient’s assertiveness, the structure of the business aspects of the care, the course of the day (visits on the ward, outpatient, who was before you, etc.), and so on. All these forces come together in the caregiver, and although we can quite predict which attentiveness will occur from that, it fundamentally escapes our understanding which seems to be based on ‘producing’ attentiveness.

The idea of an existence of inexplicabilities is consistent with the care ethical assumptions that good care is always relational, context-bound, and therefore unique. From the interaction among people and between them and the environment, things may become visible that previously were not. This requires some openness in the attentiveness of the caregiver. By having open attention, i.e. attention that is not completely framed but receptive to what may emerge, a relationship may be created that is wider than just functional, allowing what really matters to pop up.

In current discussions on healthcare that must be attentive to patients, the emphasis is on understanding patients by obtaining as much insight into their experiences as possible (Department of Health report 2010). Consequently, more and more studies focus on patient experience and lifeworlds. However, the working of emergent properties shows that this is not enough. On the one hand, caregivers gain understanding by information on patient experiences. These can make them more sensitive to the various experiences of the patients they encounter. On the other hand, we also have to realize that health care professionals should not want to understand everything. Understanding also means defining or settling, and this is too static a meaning to be attentive to patients. Attentiveness should not only consist of your own active inquiry, but also by ‘receptiveness’ or the mode of ‘letting things happen’.

The emergent properties make clear that good care depends on the recognition of the indefinite. We see that attentiveness is often focused on an object, but for good care it is essential that attentiveness is open to a certain degree. Therefore, openness, or indeterminacy, should have a place in our thinking about care. Perhaps we must abandon the idea that attentiveness must always be focused on something. But how can someone be attentive without knowing what to focus on?

## Attentiveness: the indefinite as essential

In the care relationship, due to the attention, something may come into existence that is often absent or invisible beforehand. We have illustrated this with some examples. It becomes clear that being an attentive caregiver is not always about trying to determine the object of attention, i.e. attribute a fixed meaning, but rather to postpone the interpretation, or to continue interpreting. Interpreting is understood here as a process, something that is not static, but moving. Gadamer (1997) describes an ever expanding circle of understanding and interpretation in which we approach a topic with some preconceptions, or projections. These projections are then examined and revised in the face of what “the things themselves” reveal to us. Then we return to a further exploration in the light of this new understanding. In addition, the topic is understood by viewing “the whole in terms of the detail and the detail in terms of the whole” (p. 291). This dynamic movement of understanding from projection to topic to new projection, and from whole to part to whole, constitutes the hermeneutic circle of understanding and interpretation. Open attention should not only be described as actively searching. It is also a kind of waiting; a process of learning; a process of letting something come to you. This section discusses some authors commenting on this indefiniteness or openness.

Iris Murdoch, philosopher and novelist, shows in her essays from the 50s and 60s how morality is a matter of open attentiveness. For Murdoch ‘looking’, as an ‘action of attentiveness’, is a metaphor for ‘seeing’: forming a picture of the other as he really is. She illustrates this by means of a story about a mother who is not happy with her daughter-in-law, as she thinks her unpolished behaviour is not good enough for her son. However, out of courtesy, she does not show it. Consequently, because the mother does not turn away from the daughter, she does not stick with the rejection. As she continues to look at the daughter-in-law and tries to see through her unpolished behaviour, she focuses her gaze on just that part which is so difficult to see, and thus she ‘looks for the best in her’. She tries to see the daughter-in-law not ‘accurate’ in the sense of logically correct, but she tries to see her ‘right’, to do her justice. The mother is not trying to understand what she sees; she only needs to see it ‘clear’. According to Murdoch, this seeing clearly unfolds in a process of looking: in a process of ‘careful and just attention’ (1997). In this process, she is going to see other things: other conduct than the unpolished behaviour. It is a kind of looking that starts from the good in the daughter-in-law. As the mother is guided by the good, even though she only sees unpolished behaviour on the surface, she does the daughter-in-law justice.

Murdoch makes a distinction between seeing and understanding, or “seeing clearly” versus being logical and correct. This difference is also cited by Baart (2004: 55) when he writes about the Greek word “diagnoses”. In this kind of compound words, “dia” usually means something like “going through something.” “Gnos” can be translated as to know or understand. Diagnostics is the doctrine of seeing through: understanding through the things. This means not to stop at the phenomena as they appear, but look through them, with the assumption that behind or beneath the deceptive appearance, the true reality of a phenomenon lies: its essence.

Simone Weil says: ‘it is not important to understand new things, but to learn to fathom, with patience, effort and method, obvious truths with your whole being’ (1949: 223). Just like Baart, with this “fathoming” she refers to a deeper layer. Weil considers thinking—she calls it studying—as gymnastics for the attentiveness, but no more than that, because ultimately attentiveness is about something else. It is, according to Weil, about distinguishing between reality and illusion. The aforementioned “looking” is indeed a way to exercise the mind, but it is also about looking without attachment. For Weil, attentiveness is the ‘suspension of the thought and the experience and allowance of the emptiness’ (1949: 229). According to Weil, attentiveness is not a result of the will (i.e. the mode of producing’), but of a desire (i.e. the mode of ‘waiting’). This is an important nuance: attentiveness comes down to really desiring, but not to trying to accomplish it. To Weil, it is about an attentiveness that is so concentrated that the “I” does play no role. In the words of Murdoch attention is an imaginative and normative use of moral vision that burns away the selfishness of natural human desire, leaving behind the purified desire or just and compassionate love (1970).

## Attentiveness that creates

According to Murdoch and Weil, open attentiveness is about a way of seeing that ‘imagines’ love. Weil argues that when attentiveness is intense enough, it coincides with the ability of a human to “create”. This creation is relevant from the perspective of care, as care is about getting to ‘the good’ in the relationship between caregiver and patient. It is not always clear what is good for a particular patient in a particular situation. However, this may crystallize in the relationship. The caregiver’s open attentiveness can help giving shape to this good: slowly it can be imaged who he can be for the patient and what his attention should focus on.

Waldenfels (2004) also refers to this *creating attentiveness*. He states that attentiveness consists of certain types of actions and accidents (‘being given’) that must be created. These types of experiences do not exist in the world of physical things and processes, nor in the inner world of mental acts. They must be “created” by “determining what is undetermined”. Instead of intentionality joining “us” with “the world” (as per Merleau-Ponty’s phrasing), Waldenfels describes a responsivity that exists between the “order” on the one hand and the “alien” on the other. Correspondingly, his focus is on boundaries, borders and limits: on thresholds of attention, on the twilight of order, on the human as a “liminal being” (2011, pp. 8–20), and significantly, on the dia, the “between words”, as contained in the word dialog (ibid.). This applies to the doctor in the first example above: not only does he hear the words spoken by the patient, but he is also attentive to what is said “between the lines”.

Husserl also emphasizes this indefiniteness or vagueness “in between”. Creative perception means seeing and hearing something new by seeing and hearing in a new way. […] Creative attention refers to a special dimension of experience that we characterize as *pathique* and *responsive* (Husserl in Waldenfels 2004). This means no experience can exist without somebody to whom it happens, whether it may be a case of pleasure, of pain, of joy or of sorrow. Vice versa there is no response without something to which or somebody to whom it responds. ‘What takes place here on a deeper level precedes and exceeds every sort of sense and rule; it goes beyond intentionality and regularity. Whatever strikes or affects us does not possess any sense or follow any rule in advance, it only obtains a certain sense and a certain regularity by the creativity of our answers’ (ibid). Husserl does not see creation as something like a pure creation which would transfer us straight into a world of imagination. On the contrary, ‘creative responses transform and deform given forms in a way similar to how the Revival re-created the imagery of Greek-Roman antiquity’ (ibid.).

To open up this deeper dimension, Waldenfels argues we need a special kind of responsive attentiveness that interrupts the progress of the natural experience and gives up what we take for granted. This does not lead us to what our experience means, but rather to what our experience is responding to. This applies to the doctor in the second example above. In letting the patient play with him a little, and in not being able to explain what he is doing and for what reason, he leads us to what his experience is responding to.

Merleau-Ponty (1945) writes about attentiveness as a transformative act. According to Merleau-Ponty, attentiveness can bring about a transformation of the mental field by adhering to turning points. Unlike a single mention of anything due to the importance of the subject, or the surprising nature of the object, Merleau-Ponty understands attentiveness as a new way of being present to things. Attentiveness is a transformation on the way it is aware of something. ‘In attention, consciousness can become attentive and attend to being-in-the-world, to the presence of the world and not merely to the present world at hand’ (Sá Cavalcante Schuback 2006: 138). This transformative attentiveness then points at a rediscovery of things.

Verhoeven calls this ‘wondering’. Rather than understanding this as something unexpected coming to us that we had never experienced that way before, he claims that wonder creates a transformation in which the previously experienced things can be seen in a new light. Attentiveness in the meaning of ‘wonder’ is a respite from ingrained patterns of perceiving, naming, thinking, and acting. Attentiveness therefore means a transformation in perception and knowledge.

In his book on the art of hunting, the Spanish philosopher Ortega y Gasset creates a type of phenomenology he calls *thehunter*’*s attention*. He describes hunting as letting go of a focus. A hunter is someone who has learnt how to wait. The hunter has learnt to expect the unexpectable. This vision resembles Simone Weil’s: the hunter’s attention is not connected to anything that’s already there either, nor is it the ability to respond quickly to surprising occurrences. For the hunter, attentiveness is related to the open indeterminacy of imminent events (Ortega y Gasset 1960). That openness is odd, because openness can only catch our attention when we divert our attention from the indicated objects. It is precisely at the moments when attention focused on fixed points is interrupted that open attention has a chance to break through.

In sum, attentiveness is neither a collage of outer mechanisms and internal acts, nor a scale leading gradually from passivity to activity. On the contrary, it is carried on by a radical kind of passivity. This sort of passivity proves to be more than the mere counterpart of our own activity and more than a diminished degree of activity. Responding means to start from elsewhere, from what is alien to us. While responding to the other’s appeal we step outside ourselves.

## Attentiveness and mindfulness

This sort of passivity is cultivated in the Buddhism-oriented movement of *Mindfulness*. *Mindfulness* recognizes the double event of attention (being affected by and responding to) and can be described as a non-judgmental presence in the here and now. It is used both in a psychotherapeutic context (e.g. in the treatment of anxiety and stress), and in a more ideological context (meditation inspired by Buddhism). *Mindfulness* has also been described as an art of living marked by an aversion to the hasty life. These forms of *mindfulness* are particularly aimed at the ego, the self and therefore their relevance for hospital care mainly lies in self-care. Research has not clarified whether practising *mindfulness* leads to, for instance, more open attention for care recipients or to paying more attention when carrying out certain tasks. This could still be the case, because practising *mindfulness* can result in concentration, which in turn will result in insights (Hanh 2009). When it comes to *mindfulness*, two forms of concentration can be distinguished: the active form and the selective form. The active concentration exists in the here and now and is open to anything that presents itself. When selective concentration is practiced, the attention is persistently focused on one object of choice. This concentration creates an intense type of presence, which results in stability and calmness. The higher the level of meditative concentration, the more insights are achieved. Another important aspect of *mindfulness* is attentiveness. This can be focused on our bodies, our feelings, our minds, and the object of our minds. Just like concentration, the attentiveness is focused on the present moment, enabling us to make contact with things or other people. This leads not only to understanding, but also to new perspectives and transformation (ibid). Themes of a similar nature can be found in Benedictine spirituality (Casey 2005; Grün 2006; Derkse 2003). Both *mindfulness* and these Christianity-oriented ideas on living in attention are about permanently practising ‘the respectful receptivity of the infinite other’ (Baart 2008: 9).

## Attentiveness and unknowing

When it comes to care, the point of the double character of attentiveness is that the caregiver, despite his lack of understanding, does not turn the gaze away but keeps watching. Attentiveness as described above is open and, to a greater or lesser extent, searches for an understanding of what the proper focus must be. It is both active and passive. Some forms of open attention do not even seem to pursue any understanding at all but advocate a kind of “un-knowing”:

Knowing is wonderful, but it is just a guiding means. Unknowing is a condition of openness. This unknowing in the intersubjective space of two people or people of two cultures allows others to be. This art of unknowing may enable a nurse to understand, with empathy, the actual essence of the meaning an experience has for a patient. This pattern of unknowing focused herein on the intersubjective whole between patient and nurse is applicable as well to learning in a more formal sense. To be open to learning one needs to posture oneself in a position of unknowing to hear a colleague, a teacher, a student. To provide and find openness is to be able to say, “I never thought about it that way,” and at once experience the wonderment of coming upon an “unknown” (Munhall 1993: 125).

Open attentiveness means a certain unknowing, a kind of swinging with what happens and a loosening of the reins, with the assumption that the unseen will show. This seems to be against the rules of medicine, in which everything must be monitored and controlled. In some situations however, good care requires unknowing attentiveness that is not focused on results or goals.

As stated before, we cannot always fully understand patients. In practice, not understanding often means that caregivers direct their attention toward something else, something they are able to place. However, the above literature shows that attentiveness is not necessarily connected to grasping the other’s point of view. We do not have to understand patients in order to be attentive to them.

Like patients, no experience is the same. Describing an experience is difficult. Once we give words to the experience, we have to deal with an inevitable loss of meaning: “When you say the word flower, you have already lost the bouquet,” the poet Mallarmé writes. Words like to stick, and do not allow escape. Rational, descriptive knowledge describe reality so much that there are also aspects that escape this described reality; there is too much firmly fixed to allow for a more comprehensive meaning (Bos 2011).

It seems to go against the current organization of health care, since everything needs to be determined precisely, but good care cannot do without indeterminacy. We should not just focus on the patient experience, we should also realize that we cannot grasp it fully, and create a kind of reservation. At the same time, this reservation should have a place in our thinking and evaluating of the quality of care, and should not be stashed away.

## Discussion

How can caregivers be attentive to patients despite of the impossibility of fully grasping their experiences? This paper elaborated on attentiveness, and discussed the meaning of attentiveness defined as totally determined by an empirically made transparent, causal mechanism. It concluded that attentiveness can also be undetermined, unfixed, or pending.

Much has been written about patient-centredness, patient experiences, patient lifeworld, and so on. The claim is that these kinds of research may help caregivers becoming more sensitive to what is at stake for patients, by taking up an *emic* point of view. Of course, this is a very good idea. But there is also something else going on, which partly contradicts that: it is impossible for caregivers to fully understand patients. When it comes to attentiveness in health care, we need to thematize this impossibility as well: the emptiness, the lack of understanding.

Strikingly, Blanchot (1997) writes that some experiences ask for “inattentiveness”—negligence and absentmindedness—rather than attentiveness. His concern is a special kind of inattentiveness. Not an insensitivity that only betrays contempt, because such insensitivity might just be about an “I”, who imagines he is the centre of the universe. The inattentiveness Blanchot writes about is more passive, less calculating, and less aggressive. In this carelessness it is not the “I” that is key. On the contrary, the “I” is exposed to a passion for the passive, for not-doing, for negligence. This passion for the passive is characterized by the fact that, as Blanchot puts it, the eyes remain open without them seeing (‘les yeux ouverts sans regard’). The “I” disappears. There is no one who wants to grasp the world anymore.

We think the literature that presents patient experiences and thereby claims to provide insight or understanding, partially falls prey to the problem that is precisely identified by the authors. The more we try to get a grip on the experiences of patients by translating it into ‘knowledge about patients’, the more it will actually escape our understanding. If we want to do justice to the experience of (sick) people, to their unique experiences, we might have to focus on the impossibility of grasping the other’s point of view, rather than on the urge for understanding.

The growing interest in research into the experience of patients is not the same as being attentive to patients, as long as the research is seen as a tool to be more attentive to patients in health care. Attentiveness is not something we have, and not something we can shape arbitrarily. Attentiveness has us and shapes us. We can often direct our attention, but it will always be characterised by a certain level of uncontrollability as well. Our attention surpasses our own projects, just as it surpasses the various techniques and practices by which our attentive behaviour is modelled.

## Conclusion

Does the current constant urge to understand the experiences of patients threaten the attentiveness to patients? This paper tries to make credible that it is not the question whether patients will still be seen in the future. There is no reason to fear that attentiveness will disappear. There is rather a danger that the desire to “make” attentiveness, will cause attentiveness to be understood as combating ignorance. As health care professionals, we must not attempt to understand patients fully. Conversely, understanding is being attentive to what comes into existence because of the fostering attentiveness; to what shows itself to the extent that attentiveness seeks the mode of ‘letting things happen’ (receptivity), and is not imposing functionality but respecting otherness. Only in this meaning, we avoid that out of the fear of being confronted with a lack of understanding, we as caregivers fill gaps with our own impressions and thereby take the position of the patient. ‘Reflecting on our own experience to understand the other is balancing between “ego-centrist” non-understanding and empathetic understanding of the other in terms of ourselves’ (Van der Geest 2007: 9). We all carry the experiences of being sick, of uncertainty and dependency, with us in our bodies, in our family ties, in our culture, and in our language. It is in those places where we experience an understanding of what cannot be understood. The desire to be attentive to others will report itself from those places. But only if we can leave room for it, and if we do not fill this space with well-defined views about what should be understood, and for what sense and benefit.

## Footnotes

1 However, sometimes it seems to be like that: e.g. when a caregiver aims to ‘give some attention’. But this is not what we mean. We do not necessarily understand the meaning of attentiveness in the same way as caregivers do.

2 The existing literature on emergence makes a distinction between strong and weak emergence (Chalmers 2001; Bedau 1997).
